# Live Recombinant *Salmonella* Typhi Vaccines Constructed to Investigate the Role of *rpoS* in Eliciting Immunity to a Heterologous Antigen

**DOI:** 10.1371/journal.pone.0011142

**Published:** 2010-06-18

**Authors:** Huoying Shi, Javier Santander, Karen E. Brenneman, Soo-Young Wanda, Shifeng Wang, Patti Senechal, Wei Sun, Kenneth L. Roland, Roy Curtiss

**Affiliations:** Center for Infectious Diseases and Vaccinology, The Biodesign Institute and School of Life Sciences, Arizona State University, Tempe, Arizona, United States of America; Columbia University, United States of America

## Abstract

We hypothesized that the immunogenicity of live *Salmonella enterica* serovar Typhi vaccines expressing heterologous antigens depends, at least in part, on its *rpoS* status. As part of our project to develop a recombinant attenuated *S*. Typhi vaccine (RASTyV) to prevent pneumococcal diseases in infants and children, we constructed three RASTyV strains synthesizing the *Streptococcus pneumoniae* surface protein PspA to test this hypothesis. Each vector strain carried ten engineered mutations designed to optimize safety and immunogenicity. Two *S*. Typhi vector strains (χ9639 and χ9640) were derived from the *rpoS* mutant strain Ty2 and one (χ9633) from the RpoS^+^ strain ISP1820. In χ9640, the nonfunctional *rpoS* gene was replaced with the functional *rpoS* gene from ISP1820. Plasmid pYA4088, encoding a secreted form of PspA, was moved into the three vector strains. The resulting RASTyV strains were evaluated for safety *in vitro* and for immunogenicity in mice. All three RASTyV strains were similar to the live attenuated typhoid vaccine Ty21a in their ability to survive in human blood and human monocytes. They were more sensitive to complement and were less able to survive and persist in sewage and surface water than their wild-type counterparts. Adult mice intranasally immunized with any of the RASTyV strains developed immune responses against PspA and *Salmonella* antigens. The RpoS^+^ vaccines induced a balanced Th1/Th2 immune response while the RpoS^−^ strain χ9639(pYA4088) induced a strong Th2 immune response. Immunization with any RASTyV provided protection against *S. pneumoniae* challenge; the RpoS^+^ strain χ9640(pYA4088) provided significantly greater protection than the ISP1820 derivative, χ9633(pYA4088). In the pre-clinical setting, these strains exhibited a desirable balance between safety and immunogenicity and are currently being evaluated in a Phase 1 clinical trial to determine which of the three RASTyVs has the optimal safety and immunogenicity profile in human hosts.

## Introduction

Vaccines based upon live, attenuated pathogens were originally developed to prevent infection by homologous pathogens. The success of these vaccines ((BCG, oral polio (Sabin), MMR and Ty21a)) raised the possibility that a live, attenuated pathogen could be used to stimulate immunity not only against itself, but also heterologous pathogens by expressing foreign antigens. This idea is particularly attractive for pathogens that require a complex or mucosal immune response for protection, since other vaccination strategies are not as effective at stimulating these kinds of immune responses. The success of the chemically attenuated *S*. Typhi Ty21a vaccine [1] indicated the feasibility of developing rationally attenuated, highly immunogenic live *S*. Typhi vaccines.

Early success with live attenuated homologous bacterial vaccines led to the development of strategies to modify the vaccine strains for use as vectors to deliver a variety of protective antigens via the mucosal route. This is particularly advantageous for pneumococcal protein antigens as delivery via the mucosal route elicits secretory as well as serum antibody responses, resulting in protection against carriage as well as systemic disease [2], [3], [4]. Recombinant attenuated *Salmonella enterica* serovar Typhimurium vaccines (RASVs) expressing the pneumococcal antigen gene *pspA* have been constructed and demonstrated to induce protective immunity in mice against challenge with virulent *S. pneumoniae* [5], [6]. Translating what works in *S*. Typhimurium for mice into a *S*. Typhi-based vaccine for humans has led to mixed results. Although Δ*galE* [7], Δ*aroC*, *ΔaroD* [8], Δ*cya Δcrp* [9] and Δ*phoPQ* [10], [11] mutants of *S*. Typhimurium were completely attenuated in mice and induced protective immunity to challenge with wild-type *S*. Typhimurium, *S*. Typhi Δ*galE* [12], Δ*aroC* Δ*aroD* and Δ*cya* Δ*crp* [13] mutants were not adequately attenuated and caused significant reactogenicity in humans. It thus became necessary to devise new strategies to overcome reactogenicity issues. For example, the strains such as (Δ*cya Δcrp-cdt*) [14], [15], CVD908-*htrA* [16] and an Δ*aroC* Δ*ssaV S*. Typhi Ty2 [17] are reported to be safe and immunogenic in humans. However, a *phoP* mutant of *S*. Typhimurium, completely attenuated in mice [10], led to development of a safe and immunogenic Δ*phoPQ S*. Typhi vaccine strain [18].

Although RASVs have been successfully used to express recombinant antigens and induce protective immunity in animals [19], recombinant attenuated *S*. Typhi vaccines (RASTyV; vaccine strains synthesizing heterologous antigens) have not been sufficiently effective in human studies to justify commercial development [20]. There are several reasons for the lack of success using live *S*. Typhi vectors in humans. The major difficulty has been in establishing a balance between eliciting a strong immune response to the vectored antigen and reactogenicity. Non-reactogenic strains are often hyperattenuated and induce poor immune responses, even to *Salmonella* antigens [14], [18]. It is likely that the recombinant *S*. Typhi strains do not colonize lymphoid tissues such as the gut-associated lymphoreticular tissues (GALT), mesenteric lymph nodes and spleen to a sufficient level to stimulate a robust immune response against the foreign antigen. The host immune system may also be “distracted” by the *Salmonella* carrier, mounting a strong response against *Salmonella* antigens instead of the vectored antigen.

Recently, we have begun to understand another possible reason for the poor results with live RASTyV strains expressing protective antigens from a diversity of pathogens. It was reported that *rpoS* mutations attenuate *S*. Typhimurium [21] and this led shortly thereafter to the demonstrated role of RpoS in regulating genes on the *S*. Typhimurium virulence plasmid [22], [23]. *S*. Typhimurium strains with *rpoS* mutations have markedly diminished abilities to colonize the Peyer's patches in mice [24]. Virulence plasmid-cured *S*. Typhimurium strains colonize Peyer's patches as well as wild-type strains [25], indicating that chromosomal genes controlled by *rpoS* must be of critical importance for Peyer's patch colonization. We thus theorized that *rpoS* mutations not only attenuate *Salmonella* but also likely lessen their immunogenicity. This fits with past experiences when it was reported that *S*. Typhi Ty2, which had been used by most groups constructing RASTyV strains to evaluate in humans, was an *rpoS* mutant [26]. When administered at high doses, *S*. Typhimurium *rpoS* mutants can protect against homologous, but not heterologous challenge [26], [27]. In our own unpublished studies, we examined the immunogenicity of *rpoS* mutants. In a dose response study, we found that introduction of an *ΔrpoS* mutation reduces the protective efficacy of a live *S*. Typhimurium strain (unpublished results). Introduction of a regulated *rpoS* expression cassette [28] into a recombinant attenuated *S*. Typhimurium vaccine strain results in a substantial reduction in the immune response directed against the vectored antigen (unpublished results). Therefore, we postulated that an RpoS^+^ *S*. Typhi RASV strain would be more effective at inducing protective immune responses to recombinant antigens than an RpoS^−^ *S*. Typhi (e.g. Ty2) vaccine strain.

Based on our current knowledge about the RpoS phenotypes of *S*. Typhi strains, it can be inferred that attenuated RpoS^+^ *S*. Typhi strains are more virulent in humans than equivalent RpoS^−^ *S*. Typhi strains with the same attenuating mutations [13]. Use of RpoS^+^ *S*. Typhi antigen delivery vectors will therefore necessitate degrees of attenuation in excess of what is necessary to attenuate *S*. Typhi Ty2 strains for humans, but these means of attenuation must not compromise immunogenicity.

*S*. Typhimurium infection in mice is often used as a model of typhoid fever, due to the host-restriction of *S*. Typhi and the absence of a small animal model to evaluate typhoid fever caused by *S*. Typhi. We have recently developed a number of new systems in *S*. Typhimurium designed to enhance the safety and immunogenicity of RASVs, including regulated delayed *in vivo* attenuation [28], [29], regulated delayed antigen synthesis [30] and regulated cell lysis [31]. We have also modified antigen, repressor and plasmid vector sequences to enhance transcription and translation efficiencies to maximize protective antigen synthesis *in vivo*. In addition, we have also explored other means to enhance immunogenicity and safety, including the *ΔsopB* mutation, which reduces *Salmonella*-induced fluid secretion in the intestines [32], [33], [34] and enhances the immunogenicity of a vectored antigen [35]. Many of these features were combined to create *S*. Typhimurium strain χ9558 [34]. In addition, this strain also has a *ΔasdA* mutation, allowing use of the antibiotic resistance-free Asd^+^ balanced-lethal plasmid maintenance system [36]. When complemented with an Asd^+^ plasmid that directs expression of the pneumococcal gene *pspA*, χ9558 has been shown to be safe, immunogenic and capable of eliciting an immune response that protects against challenge with virulent *Streptococcus pneumoniae*, in both adult [37], [38] and infant [34], [39] mice.

*S. pneumoniae* is a formidable bacterial pathogen, causing disease with high morbidity and mortality even in regions where antibiotics are readily available. *S. pneumoniae* is estimated to kill 1–2 million children under the age of 5 years each year in developing countries, accounting for 20–25% of all deaths in this age group [40]. Antibodies to pneumococcal capsular polysaccharides can protect against fatal infection, but this protection is serotype specific. Current vaccines based on capsule polysaccharides therefore provide coverage only for the specific serotypes included in the vaccine. There are 91 distinct capsular serotypes of *S. pneumoniae* [41], [42], and geographic differences in serotype prevalence have resulted in suboptimal protection in many countries. Recent reports have shown that although carriage of vaccine serotypes was reduced in immunized individuals, the vacated niche was promptly occupied by non-vaccine serotypes [43], [44], [45]. This “replacement carriage” has translated into a significant increase in cases of invasive disease caused by non-vaccine serotypes in conjugate vaccine recipients [46]. Because of these concerns, we focused our attention on developing a vaccine based on pneumococcal proteins, such as PspA, that contribute to virulence and are common to all serotypes [47].

In this work, we constructed three new recombinant attenuated *S*. Typhi vaccines (RASTyV) derived from *S*. Typhi Ty2, its RpoS^+^ derivative [48] and ISP1820 expressing *pspA* from an Asd^+^ expression plasmid. We introduced a constellation of mutations nearly identical to those present in *S*. Typhimurium strain χ9558 [34], [38]. In particular, two mutations, Δ*pmi* and *ΔtviABCDE*, affecting synthesis of two major *S*. Typhi surface antigens, O-antigen and Vi capsule, respectively, were included in an attempt to reduce the host immune response to *Salmonella*, and, hopefully, to enhance the immune response to the vectored antigen, PspA. We evaluated each strain for safety, persistence in the environment and in human blood and immunogenicity in mice.

## Materials and Methods

### Ethics statement

All research involving human participants was conducted as per Protocol #0804002872, approved by the Arizona State University Institutional Review Board. Informed consent was obtained from all participants in accordance with the Declaration of Helsinki. The Arizona State University Institutional Animal Care and Use Committee approved all animal procedures.

### Strains, plasmids and culture conditions

The strains used in this study are described in Table 1. *S*. Typhi strains Ty2 and Ty21a were kind gifts from Lou Baron at WRAIR. Asd^+^ plasmids and suicide plasmids are listed in Table 2. Cultures were routinely grown at 37°C in LB broth [49] or LB agar. Strains were stored in either peptone-glycerol or animal-free phytone-glycerol (1% peptone or phytone (Difco, Detroit, MI, USA) and 5% glycerol (Fisher Scientific Inc., Pittsburgh, PA, USA). Nutrient broth (NB) and agar (Difco), MacConkey agar (Difco), 3XD plates [50] supplemented with 50 µg/ml of 5-bromo-4-chloro-3-indolyl-phosphate (Roche, Indianapolis, IN, USA), Kornberg agar [51], chrome azurol S (CAS) plates [52], and yeast congo-red agar [53], were used for routine phenotype corroboration. When required, media were supplemented with chloramphenicol (Cm; 25µg/ml), tetracycline (Tet; 12.5 µg/ml), 2, 6-diaminopimelic acid (DAP; 50 µg/ml), L-arabinose (0.05% or 0.2% wt/vol), D-mannose (0.2% wt/vol), D-lactose (1% wt/vol), D-maltose (1% wt/vol), sucrose (5% wt/vol), L-cysteine-HCl (22 µg/ml), or L-tryptophan (20 µg/ml). Tetrathionate broth (Difco), with or without supplements, was used for enrichment of *S*. Typhi from animal tissues. Bacterial growth was monitored spectrophotometrically and by direct plating for colony counts. KT broth is a proprietary animal-free complex medium, similar to terrific broth [54], used for rapid and high-density growth of *S*. Typhi vaccine strains. Cultures maintained under different environmental stresses were plated on MacConkey agar (Difco), and then patched onto XLT-4 agar (Difco) and Bismuth sulfite agar (Difco). Oligonucleotides were from IDT (Coralville, IA, USA) (Table 3). Restriction endonucleases were from New England Biolabs, (Ipswich, MA, USA). Taq DNA polymerase (New England Biolabs) was used in all PCR tests. Qiagen products (Hilden, Germany) were used to isolate plasmid DNA, gel-purify fragments or purify PCR products. T4 ligase, T4 DNA polymerase and shrimp alkaline phosphatase (SAP) were from Promega (Madison, WI, USA).

**Table 1:** Bacterial strains used in this study.

Strains	Genotype or relevant characteristics	Source or derivation
*E. coli*		
χ7213	*thr-1 leuB6 fhuA21 lacY1 glnV44 recA1* Δ*asdA4* Δ(*zhf-2*::Tn*10*) *thi-1* RP4-2-Tc :: Mu [λ *pir*]; Km^r^	[96]
*Salmonella enterica* Typhimurium		
χ3761	UK-1 wild type	[97]
χ8477	Δ*araE25*	[28]
χ8606	Δ*agfBAC811*	[34]
χ8650	Δ*pmi-2426*	[29]
χ8767	Δ*araBAD23*	[28]
χ8831	Δ(*gmd-fcl*)-*26*	[28]
χ9277	Δ*sopB1925*	[35]
χ9021	ΔP_crp527_ :: TT *araC* P_BAD_ *crp*	[28]
χ9034	ΔP_phoPQ107_ :: TT *araC* P_BAD_ *phoPQ*	[28]
χ9226	Δ*relA198* :: *araC* P_BAD_ *lacI* TT	[98]
χ8848	ΔP_fur33_ :: TT *araC* P_BAD_ *fur*	[28]
χ9269	ΔP_fur81_ :: TT *araC* P_BAD_ *fur*	[28]
χ8958	Δ*asdA33*	UK-1
*S*. Typhi Ty2		
Ty2	RpoS^−^	[99]
Ty21a	Ty2, *galE ilvD viaB*, phenotypically H_2_S^−^	ATCC 3345
χ8438	Ty2 RpoS^+^	[48]
χ9043	ΔP_crp527_:: TT *araC* P_BAD_ *crp*	Ty2
χ9205	ΔP_fur33_:: TT *araC* P_BAD_ *fur*	χ9043
χ9213	ΔP_phoPQ107_:: TT araC P_BAD_ *phoPQ*	χ9205
χ9288	Δ*pmi-2426*	χ9213
χ9335	Δ*(gmd-fcl)-26*	χ9288
χ9369	Δ*relA198*:: *araC* P_BAD_ *lacI* TT	χ9335
χ9416	Δ*araE25*	χ9369
χ9478	Δ*tviABCDE10*	χ9416
χ9511	Δ*agfBAC811*	χ9478
χ9580	Δ*sopB*::Cm-*SacB*	χ9511
χ9584	Δ*sopB1925*	χ9580
χ9601	ΔP_fur81_:: TT *araC* P_BAD_ *fur*	χ9584
χ9603	PphoP+	χ9601
χ9639	Δ*asd33*	χ9603
χ9640	RpoS+	χ9639
*S*. Typhi ISP1820		
ISP1820	Wild-type	[100]
χ9044	ΔP_crp527_:: TT *araC* P_BAD_ *crp*	ISP1820
χ9142	ΔP_fur33_::TT *araC* P_BAD_ *fur*	χ9044
χ9196	ΔP_phoPQ107_:: TT *araC* P_BAD_ *phoPQ*	χ9142
χ9211	Δ*pmi-2426*	χ9196
χ9214	Δ*(gmd-fcl)-26*	χ9211
χ9298	Δ*sopB1925*	χ9214
χ9327	Δ*relA198*:: *araC* P_BAD_ *lacI* TT	χ9298
χ9342	Δ*araE25*	χ9327
χ9364	Δ*araBAD23*	χ9342
χ9365	Δ*tviABCDE10*	χ9364
χ9419	Δ*agfBAC811*	χ9365
χ9421	ΔP_fur_81:: TT *araC* P_BAD_ *fur*	χ9419
χ9599	PhoP+	χ9421
χ9633	Δ*asdA33*	χ9599

**Table 2:** Plasmids used in this study.

Plasmids	Relevant characteristics	Source or derivation
pDMS197	5,612 bp, Tet *sacB oriV oriT*	[101]
pMEG375	8,142 bp, Cm, Amp, *lacZ*, R6K *ori*, *mob incP*, *sacR sacB*	[102]
pRE112	5,173 bp, Cm, *sacB oriV oriT*	[101]
pYA3433	Wild-type *rpoS* allele from *S*. Typhi ISP1820, pMEG-375	[48]
pYA3484	Δ*araBAD23*, pMEG-375	[28]
pYA3485	Δ*araE25*, pMEG-375	[28]
pYA3493	Plasmid Asd^+^; pBR *ori* β-lactamase signal sequence-based periplasmic secretion plasmid	[29]
pYA3492	Δ*agfBAC811*, pDMS197	[103]
pYA3546	Δ*pmi-2426*, pDMS197	[29]
pYA3629	Δ*(gmd-fcl)-26*, pMEG-375	[29]
pYA3722	ΔP_fur33_::TT *araC* P_BAD_ *fur*, pMEG-375	[28]
pYA3723	ΔP_phoPQ107_::TT *araC* P_BAD_ *phoPQ*, pRE112	[28]
pYA3733	Δ*sopB1925*, pMEG-375	[35]
pYA3736	Δ*asdA33*, pRE112	This study
pYA3832	ΔP_crp527_::TT *araC* P_BAD_ *crp*, pRE112	[28]
pYA3879	Δ*relA198*::TT *araC* P_BAD_ *lacI*, pRE112	Lab collection
pYA4009	Δ*tviABCDE10*, pRE112	[48]
pYA4088	852-bp DNA encoding the α-helical region of PspA aa 3–285 in pYA3493	[30]
pYA4181	ΔP_fur81_::TT *araC* P_BAD_ *fur*, pMEG-375	[28]
pYA4491	ΔTT *araC* P_BAD_::P_phoP_, pRE112	This study

**Table 3:** Oligonucleotides used to verify the genotype of the RASTyV strains. F: forward primer; R: reverse primer.

Deletion/insertion	Oligonucleotides
Δ*araBAD1923*	F 5′-ACATGCATGCGGACGATCGATAAR 5′-CGGGATCCTGGTAGGGAACGAC
Δ*araE25*	F 5′-GACTGCATGCATGGTGTTGGTACAR 5′-CGGGATCCCATAGCGGTAGATG
Δ*agfBAC811*	F 5′-GCACTGCTGTGGGTTGAAATAGR 5′-CGGCGTGAGTAGAAATATCG
Δ*pmi-2426*	F 5′-GGGGGTACCTTCGGCGACGGAAACATGTTCGCTR 5′-GGGGGCTCGCCGCGCTGGTAGTTTTGATAACTTAA
Δ*(gmd-fcl)-26*	F 5′-TCCCCCGGGCAAAATATTGTATCGCTGGR 5′-GCACGCATGCTCAGGCAGGCGTAAATCGCTCT
ΔP_fur33_::TT *araC* P_BAD_ *fur*ΔP_fur81_::TT *araC* P_BAD_ *fur*	F 5′-ACATGCATGCTGTGACTGGGATGACTTCTTCCCGR 5′-TCCCCCGGGCACTTTTCCGCAATCAAGGCAG
ΔP_phoPQ107_::TT *araC* P_BAD_ *phoPQ*	F 5′-TGCGAGCTCCGATGTGGAATGGCTTCGTCACF 5′-ACATGCATGCGCAAACAAACTGCCGGTTTCCCCGC
Δ*sopB1925/sigD*	F 5′-ACATGCATGCGGCATACACACACCTGTATAACAR 5′-TTCCCCCGGGGCAGTATTGTCTGCGTCAGCG
Δ*asdA33*	F 5′-TGCTCTAGATGTGCATGGCAATCGCCCAACR 5′-TCCCCCGGGTATCTGCGTCGTCCTACCTTC
ΔP_crp527_::TT *araC* P_BAD_ *crp*	F 5′-ACATGCATGCATCTCCATCGGACTCGGCGCTTTR 5′-TGCGAGCTCCAGAATATCCGGGTTGACCTG
Δ*relA*::TT *araC* P_BAD_ *lacI*	F 5′-CCCAAGCTTGAGCTCGAGGGCGTTCCGGCGCTGGTAGAAR 5′-CGGGTACCCCAGATATTTTCCAGATCTTCAC
Δ*tviABCDE10*	F 5′-ACATGCATGCGAACGGTATTACTGTCAGTCACAAGR 5′-TCCCCCGGGCAGATTATTTCAAATACGATTAGG
ΔTT *araC* P_BAD_::P_phoP_	F 5′-TGCGAGCTCCGATGTGGAATGGCTTCGTCACF 5′-ACATGCATGCGCAAACAAACTGCCGGTTTCCCCGC

### Construction of *S*. Typhi strains with defined unmarked chromosomal insertion/deletions

**Figure 1:**
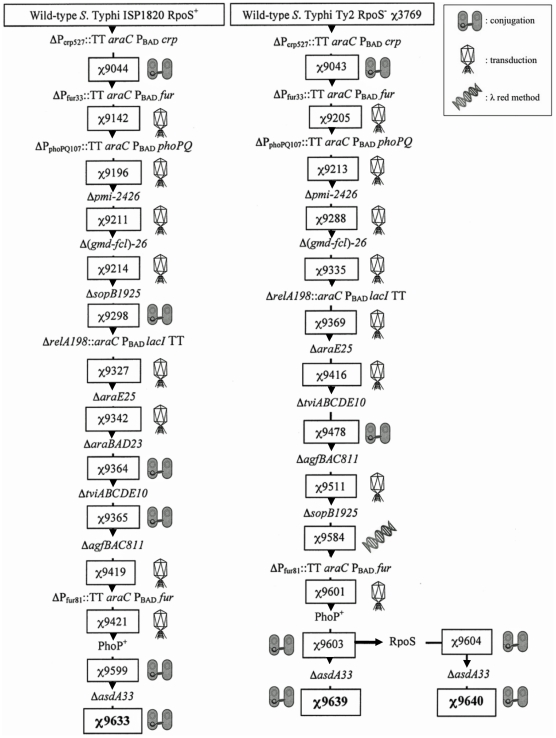
Genealogy of the RASTyV strains construction. This figure illustrates the steps used to construct *S*. Typhi vaccine vector strains χ9633, χ9639 and χ9640. Beginning at the top of the figure with parent strains ISP1820 and Ty2, individual mutations or other genetic modifications were introduced using suicide plasmids introduced either by conjugation or transduction. In one case, the mutation was introduced using the λ red method.

We used several means to generate defined deletion and deletion-insertion mutations leaving behind no DNA scars or antibiotic-resistance markers by using suicide plasmid-based technologies [55], [56] and a two-step recombination method [57]. In all cases, we determined the DNA sequence of the flanking regions used to construct chromosomal mutations. We made insertions of *araC* P_BAD_ into the chromosome by cloning *araC* P_BAD_ between DNA regions flanking the desired insertion point [28]. The *araC* P_BAD_ cassette we used includes transcription terminator (TT) sequences to preclude *araC* transcription reading into adjacent genes and interfering with their function [28]. By the same logic, a TT sequence was included downstream of *lacI* in the *ΔrelA198* deletion/insertion. We used these methods to generate most of the deletion/insertions in *S*. Typhimurium. Mutations were moved from *S*. Typhimurium (Fig. 1) into *S*. Typhi strains by P22HT*int* transduction [58]. Suicide vectors were used to introduce some mutations into *Salmonella* strains by conjugation with *E. coli* χ7213 [48].

### Characterization of *S*. Typhi strains *in vitro*

All the *S*. Typhi constructions were characterized for type I fimbriae production in static LB broth cultures [59] by yeast agglutination [60] and for motility in motility medium (bioMériux, Marcy I'Etoile, France). Lipopolysaccharide (LPS) profile was evaluated by sodium dodecyl sulfate-polyacrylamide gel electrophoresis then visualized by silver staining [61]. Plasmid profiles were verified by alkaline lysis and agarose gel (0.5%) electrophoresis [62]. Biochemical profiles were determined in the presence and absence of 0.2% arabinose using the API 20E system (bioMériux). The presence of Vi, O and flagella antigens were evaluated by agglutination assays. The presence or absence of RpoS was determined by addition of hydrogen peroxide to cultures to detect activity of the RpoS-dependent catalase, KatE [63], [64], and by glycogen accumulation when streaked on Kornberg glycogen indicator agar [65]. The presence of each chromosomal mutation was verified by PCR using the appropriate DNA primers (Table 3). Stability of Asd^+^ plasmids in vaccine strains was determined as described [66].

### SDS-PAGE and immunoblot analyses

To evaluate arabinose dependent regulation of gene expression, strains were grown in NB medium with 0.05% arabinose at 37°C with aeration. When the culture reached an OD_600nm_ of 0.8, it was diluted 1∶10 into fresh NB without arabinose and grown to an OD_600_ of 0.8. This process was repeated 6 times (∼20 generations). One ml of culture was collected from each passage and prepared for western blot analysis [54]. Total protein was normalized, samples were separated on a 10% (wt/vol) sodium dodecyl sulfate-polyacrylamide gel and transferred onto nitrocellulose membranes. Fat-free milk powder dissolved in phosphate buffered saline (PBS) (5%, wt/vol) supplemented with 0.05% Tween 20 (PBS-T) was used for blocking. The membrane was incubated with an appropriate anti-rabbit polyclonal antibody (1∶10,000), anti-Fur, anti-Crp, and anti-LacI [28], anti-PspA [30], anti-GroEL (Sigma, St. Louis, MO, USA) or anti-mouse monoclonal antibody (1∶1,000) anti-RpoD (Neoclone, Madison, WI, USA) for 1 h at room temperature, washed three times with PBS-T, and then incubated with a 1∶10,000 dilution of alkaline phosphatase-conjugated anti-rabbit immunoglobulin G (IgG) (Southern Biotech, Birmingham, AL). Color was developed with nitroblue tetrazolium and 5-bromo-4-chloro-3-indolylphosphate (BCIP) (Sigma).

### *Salmonella* subcellular fractionation

Cultures were grown in NB to an OD_600_ of 0.6 and centrifuged at 5,800×*g* for 10 min. Periplasmic fractions were prepared by a modification of the lysozyme-osmotic shock method [67] as previously described [6]. The supernatant fluid was saved for analysis of secreted proteins. Equal volumes of outer membrane proteins (OMPs), periplasmic, cytoplasmic, and supernatant fractions and total lysate samples were separated by SDS-PAGE for western blot analysis.

### Deoxycholate sensitivity assay

RASTyV strains and wild-type *Salmonella* Typhi strains were grown in KT broth to a cell density of approximately 1×10^9^ CFU/ml (late log phase). The growth media for the RASTyV strains were supplemented with 0.2% arabinose and 0.2% mannose. Cultures were harvested by centrifugation at 2500×*g* for 20 min at room temperature. Cells were resuspended in PBS and allowed to acclimate for one hour. Following acclimation, the cells were pelleted and the PBS was removed. Cells were transiently exposed to sodium deoxycholate in PBS (0, 0.1, 0.25, 0.5, 1.0, 2.5 and 5.0%) for 2 h at 37°C (final concentration 1×10^9^ CFU). The number of viable cells after deoxycholate treatment was assessed by duplicate plating on LB agar+0.2% arabinose. This experiment was repeated three times.

### Survival of *Salmonella* strains in chlorinated water, surface water and in raw sewage

Chlorinated water was prepared by dissolving a crushed chlorine pool tablet into distilled water to a final concentration of 4∼5 ppm (chlorine concentration was determined by the SenSafe chlorine testing kit (Industrial Test Systems, Rock Hill, SC, USA)). Chlorinated water was used within 10 min of preparation. Frozen stocks were thawed at 37°C, and then residual phytone-glycerol was removed by washing cells with PBS. Cells were suspended in 1 ml of PBS to a concentration of 1±0.2×10^9^ CFU/ml. The actual titer was measured by plating serial dilutions onto LB agar+0.2% arabinose. 1×10^9^ CFU of cells were diluted into 19 ml of chlorinated water, and viability was assessed by plating at 10, 30 and 60 min after the addition of chlorine. Samples were prepared in triplicate and plated in duplicate at all time points.

Raw sewage samples were obtained from the Kyrene Water Reclamation Facility in Tempe, AZ and were used within one week of collection. Untreated surface water samples were obtained from Tempe Canal in Tempe, AZ and were used within one week of collection. The sewage was diluted 1∶1000 in PBS prior to use to reduce the number of background organisms to <1×10^5^ CFU/ml. *S*. Typhi wild-type and RASTyV strains from phytone-glycerol frozen stocks were thawed, and the cells washed with PBS, then inoculated into triplicate 20 ml surface water or sewage samples to a concentration of 1×10^8^ CFU/ml. Samples were incubated at ambient temperature (21–25°C) for the duration of the assay. Viability of the *Salmonella* strains was assessed on days 0, 1, 3, 7 and 10 after inoculation by plating on MacConkey agar+1% lactose and % mannose. For untreated surface water cultures containing wild-type ISP1820 or χ9633(pYA4088), brilliant green agar (Difco) containing 0.2% arabinose and 0.2% mannose was used in place of MacConkey agar. Each sample was plated in duplicate for each time point. Plates were compared to pure cultures streaked on identical media. All colonies suspected to be *Salmonella* were patched onto Bismuth-sulfite, XLT-4 and MacConkey agar+1.0% maltose+/−0.2% arabinose for verification. The limit of detection for this assay was 1×10^4^ CFU/ml.

### Human Subjects

Individuals who had received a vaccine directed against *S*. Typhi were excluded from participating in the study. Up to 50 ml of blood was collected from individual volunteers by venipuncture and treated with sodium heparin to prevent coagulation [68]. Samples were used as whole blood or processed to isolate monocytes within 24 h of collection.

### Survival of *Salmonella* strains in whole human blood and in human peripheral monocytes

RASTyV strains containing pYA4088, wild-type *Salmonella* Typhi strains and Ty21a were grown in KT broth to a cell density of approximately 1×10^9^ CFU/ml (late log phase). The growth media for the vaccine strains were supplemented with 0.2% arabinose and 0.2% mannose. Ty21a was grown in the presence of 0.05% galactose (this concentration was found to allow complete LPS O-antigen synthesis without reducing the strain growth rate). In some experiments, blood components were inactivated by incubation at 55°C for one hour. For survival of *Salmonella* strains in whole human blood, cells were diluted in phosphate buffered saline (PBS), pH 7.4 and inoculated into untreated or heat-inactivated fresh human blood to a density of approximately 1×10^6^ CFU/ml. Inoculated samples and uninoculated controls were incubated at 37°C for the duration of the assay. Bacterial viability was assessed by plating onto LB agar with 0.2% arabinose at 0, 3, 6 and 18 h after inoculation. The assay was performed in duplicate and was repeated 3 times using blood samples from different individuals. The limit of detection for this assay was less than 10 CFU/ml.

For intracellular survival of *Salmonella* in human peripheral monocytes, peripheral blood monocytes were isolated from human blood by Ficoll-Paque PLUS (GE Healthcare) gradient [69]. Monocytes were washed with Hank's buffered salts solution (Invitrogen, Carlsbad, CA, USA), resuspended in RPMI 1640 (Invitrogen) without serum at a concentration of approximately 5×10^5^ cells/ml and allowed to adhere to Corning Costar 24-well plates for 1 h at 37°C, 5.0% CO_2_. Non-adherent cells were removed after 1 h by washing with PBS and RPMI 1640 without serum was added to the monolayer. The bacterial invasion assay was carried out essentially as described [70]. *Salmonella* strains were suspended in RPMI 1640 at a concentration of approximately 5×10^6^ CFU/ml and allowed to invade the human monocytes for 1 h at 37°C in 5% CO_2_ (MOI of 10∶1). After one hour, cells were treated with 100 µg/ml gentamicin to kill extracellular bacteria. The number of intracellular bacteria was assessed at 1, 4 and 24 h after inoculation. Monocytes were lysed with 0.1% sodium deoxycholate for 5 min and samples were serially diluted in PBS and plated on LB agar+0.2% arabinose. This assay was performed in duplicate and was repeated three times using cells isolated from different individuals.

### Sensitivity of RASTyV strains to guinea pig serum complement

The complement sensitivity assay was carried out essentially as described [71]. Briefly, RASTyV strains with pYA4088 and wild-type *Salmonella* Typhi strains were grown in KT broth to a cell density of approximately 1×10^9^ CFU/ml (late log phase). The growth media for the RASTyV was supplemented with 0.2% arabinose and 0.2% mannose. Cells were diluted to approximately 1×10^6^ CFU/ml in PBS and were exposed to 22% purified guinea pig complement in PBS (Calbiochem, San Diego, CA, USA) in the presence or absence of Group D_1_ LPS O-antigen antibody (BD Bioscience, Franklin Lakes, NJ, USA). Reactions were incubated 3 h at 37°C. Complement-resistant cells were enumerated by plating on LB agar+0.2% arabinose. The assay was conducted in duplicate, and was repeated a minimum of 3 times for each strain.

### Mice

Female and male BALB/c mice, 6 to 7 weeks old, were obtained from the Charles River Laboratories. Mice were acclimated for 7 days after arrival before starting the experiments. Newborn mice (less than 24 hours old) were obtained from pair-wise mating of female and male BALB/c mice.

### Distribution of *Salmonella* bacteria in newborn mice

Groups of newborn mice were orally inoculated with 10 µl containing 1×10^9^ CFU of *Salmonella* vaccine strains, wild-type strains, or Ty21a. Mice were euthanized and necropsied at various times. Spleen, liver, and whole intestines were collected on days 3 and 7 post-infection. Tissues were weighed and homogenized in a final volume of 1 ml buffered saline with gelatin (BSG) [72] and serial dilutions plated onto MacConkey agar plates containing 1% lactose, and with or without 0.05% arabinose and 0.2% mannose to determine the number of viable bacteria. Plates were incubated at 37°C for at least 18 h. We also used 900 µl of homogenized tissues to inoculate 5 ml tetrathionate broth (Difco) for *Salmonella* enrichment. Samples that were negative by direct plating and positive by enrichment were recorded as 10 CFU/g. Samples that were negative by both direct plating and enrichment were recorded as 0 CFU/g.

### Immunization of mice

RASTyV vector strains harboring plasmids pYA4088 (*pspA*) or pYA3493 (empty vector) were grown in LB broth with 0.05% arabinose and 0.2% mannose overnight at 37°C as standing cultures that were diluted 1∶100 in the same medium, prewarmed, and grown with aeration (180 rpm) at 37°C to an OD_600_ of 0.8 to 0.9. Bacteria were collected by centrifugation at room temperature and resuspended in BSG to densities appropriate for the inoculation route and dose. To determine the actual CFU/dose, serial dilutions of the RASTyV strains were plated onto MacConkey agar supplemented with 1% lactose, with or without 0.05% arabinose and 0.2% mannose. Seven-week-old mice were inoculated intranasally with 1±0.2×10^9^ CFU of RASTyV vector strains carrying either the *pspA* expression plasmid pYA4088 or control plasmid pYA3493 in 10 µl. Mice were boosted with the same dose of the same strain six weeks later. Blood was obtained by mandibular vein puncture at biweekly intervals. Blood was incubated at 37°C for 60 min and the clot was pelleted by centrifugation. Serum was removed from the whole-blood samples and stored at −20°C. Vaginal-wash samples were collected at biweekly intervals and stored at −20°C as described [6]. Sera were collected 2, 4, 6 and 8 weeks after the initial vaccination and serum IgG responses to rPspA, *S*. Typhi LPS and *S*. Typhi OMPs were measured. This experiment was performed twice, with each group (8 mice) receiving approximately the same dose of vaccine, and the results from both experiments were similar and have been pooled for analysis.

### Antigen preparation

PspA protein was purified as described previously [6]. *S*. Typhi LPS was obtained from Sigma. Serovar Typhi outer membrane proteins (SOMPs) were purified from *S*. Typhi strain χ9633 as described previously [6]. The PspA-Rx1 clone was a kind gift from Susan Hollingshead at the University of Alabama at Birmingham.

### Enzyme-linked Immunosorbent Assay (ELISA)

Sera from all mice in a group were pooled for analysis. ELISA was used to measure IgG antibodies against *S*. Typhi LPS, SOMPs and rPspA in serum, IgG1 and IgG2a in serum and IgA in vaginal washes against rPspA as previously described [6], [38]. Absorbance was recorded at 405 nm using an automated ELISA plate reader (model EL311SX; Biotek, Winooski, VT). Absorbance readings that were 0.1 higher than BSG control values were considered positive [38].

### IL-4 and IFN-γ ELISPOT assays

At week 7, spleen cells were harvested from 3 mice per group. Cells from each spleen were assayed by ELISPOT in triplicate wells as previously described [73]. Briefly, PVDF membrane plates (Millipore) were washed with sterile H_2_O, and coated with 100 µl of anti-IL-4 or anti-IFN-γ mAbs (BD PharMingen, San Jose, CA, USA) at 5 µg/ml in PBS overnight at 4°C. The wells were washed with PBS and blocked with RPMI containing 10% FCS. Then, 50 µl of cell medium (RPMI-1640 supplemented with 10% FCS, 2 mM L-glutamine, 100 IU/ml penicillin, and streptomycin and 1% HEPES) and 50 µl of spleen cells (10^6^ per well) in cell medium with or without stimulation with rPspA at 5 µg/ml were added per well and incubated in the plates overnight in 5% CO_2_ at 37°C. The next day, the cell suspensions were discarded and the plates washed with PBS-T. Biotinylated anti-IL-4 or anti-IFN-γ mAb (BD PharMingen) at 5µg/ml in PBS-T with 1% FCS was added and incubated at room temperature for 2 h. After washing with PBS-T, 100 µl/well of avidin peroxidase diluted 1∶1,000 (vol/vol) in PBS-T containing 1% FCS were added, followed by incubation for 1 h at room temperature. After washing with PBS-T, 100 µl of AEC (3-amino-9-ethylcarbazole, BD Bioscience) was added per well. Spots were developed for 15 min at room temperature. Plates were dried and analyzed by using an automated CTL ELISPOT Reader System (Cellular Technology, Shaker Heights, OH, USA).

### Pneumococcal challenge

At week 10, mice were challenged by intraperitoneal injection with 1×10^4^ CFU of *S. pneumoniae* WU2 in 100 µl BSG [5]. The 50% lethal dose (LD_50_) of *S. pneumoniae* WU2 in BALB/c mice was 1×10^2^ CFU. Challenged mice were monitored daily for 15 days.

### Statistical analysis

Numerical data are presented as arithmetic means for bacterial number data and geometric mean and standard deviation in all other assays. Mann-Whitney U Test (version 5.0; GraphPad Software, Inc.) was used for comparing the distribution of *S*. Typhi in tissues of newborn mice, survival of *S*. Typhi strains in peripheral human mononuclear cells, in human blood, chlorinated tap water, raw sewage and surface water. An ANOVA (SPSS Software) analysis, followed by LSD (Least Significant Difference) method, was used to evaluate differences in antibody titer and cytokine-secreting cells response discerned to 95% confidence intervals. The Kaplan-Meier method (SPSS Software) was applied to obtain the survival fractions following i.p. challenge of intranasally immunized mice. *P*<0.05 was considered statistically significant.

## Results

### Vaccine construction and characterization

The mutations introduced into each of the parent strains, *S*. Typhi Ty2 and *S*. Typhi ISP1820, were based on several strategies including regulated-delayed in vivo attenuation [28] and regulated delayed in vivo antigen synthesis [30] worked out in *S*. Typhimurium and are comparable to the mutations in *S*. Typhimurium UK-1 strain χ9558 [34], [35], [37]. The mutations introduced into both parental strains, *S*. Typhi Ty2 and *S*. Typhi ISP1820, were *Δ*P_crp527_::TT *araC* P_BAD_ *crp*, ΔP_fur81_::TT *araC* P_BAD_ *fur*, Δ*pmi-2426*, Δ(*gmd-fcl*)-*26*, Δ*relA198*::*araC* P_BAD_ *lacI* TT, *ΔsopB1925*, Δ*agfBAC811*, Δ*tviABCDE10*, *ΔaraE25*, and *ΔasdA33*. As described below, the *ΔaraBAD23* mutation was also introduced into ISP1820 (Fig. 1). The chromosomal DNA regions flanking the point of the deletion or insertion/deletion mutations used to construct the relevant suicide vectors for the *Δ*P_crp527_::TT *araC* P_BAD_ *crp*, Δ*pmi-2426*, Δ(*gmd-fcl*)-*26*, Δ*relA198*::*araC* P_BAD_ *lacI* TT, *ΔsopB1925*, Δ*agfBAC811*, and *ΔaraE25* mutations were amplified from the *S*. Typhimurium UK-1 chromosome. The DNA flanking regions used for constructing the *Δ*P_crp527_::TT *araC* P_BAD_ *crp* and Δ*agfBAC811* mutations share 99% similarity with *S*. Typhi DNA sequence. The suicide vectors flanking region used to construct Δ*pmi-2426*, Δ(*gmd-fcl*)-*26*, Δ*relA198*::*araC* P_BAD_ *lacI* TT, *ΔsopB1925*, *ΔaraE25*, share 99–100%, 98%, 98–99%, 97–98%, 99, and 97–98% similarity with *S*. Typhi DNA sequence, respectively. The chromosomal DNA flanking regions used to construct the relevant suicide vectors for ΔP_fur81_::TT *araC* P_BAD_ *fur*, Δ*asdA33*, and Δ *tviABCDE10* mutations were amplified from the *S*. Typhi Ty2 chromosome.

**Figure 2:**
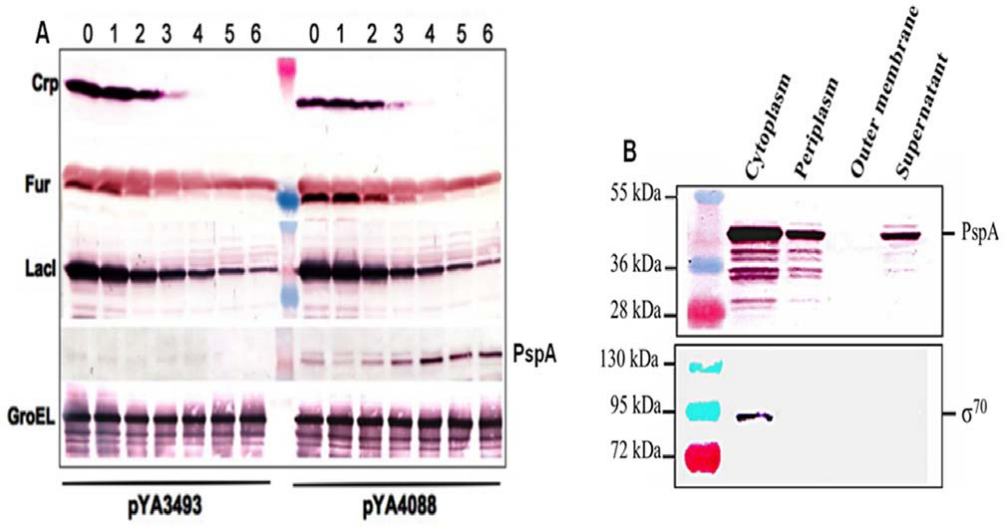
Protein expression and secretion in ISP1820 derivative χ9633 carrying either pYA4088 or pYA3493. (A) Synthesis of proteins encoded by arabinose-regulated genes. Cells were grown in NB with arabinose (Lane 0) and then diluted 1∶10 into fresh NB without arabinose every 3.3 generations. This process was repeated 6 times (∼20 generations) (Lane 1–6). Synthesis of Crp, Fur, LacI and PspA was detected by western blot. GroEL was used as a control. (B) Location of PspA in χ9633(pYA4088) cell fractions from cells grown in the absence of arabinose and detected by western blot. σ^70^ was used as a control to detect leakage of cytoplasmic contents into other fractions.

All of these mutations have been previously described [28], [29], [38], [48], with exception of Δ*asdA33*. The Δ*asdA33* defined deletion mutation encompasses a 1,104 base pair deletion including the ATG start codon but not including the TAG stop codon. The upstream flanking regions of *S*. Typhi *asdA* gene share only 55% similarity with *S*. Typhimurium. Therefore, the flanking regions used to construct Δ*asdA33* were amplified from the *S*. Typhi Ty2 genome. Although most of these mutations have been described, the details of construction for some of the mutations are described here for the first time. The *Δ*P_crp527_::TT *araC* P_BAD_ *crp*, ΔP_fur81_::TT *araC* P_BAD_ *fur*, Δ*pmi-2426* and *ΔsopB1925* mutations, attenuate *S*. Typhimurium for virulence in the oral mouse model [28], [29] (and unpublished data). In the two former mutations, the *crp* and *fur* genes are under transcriptional control of the *araC* P_BAD_ promoter. When cells are grown in broth culture in the presence of arabinose, *crp* and *fur* are expressed. After immunization, when the cells reach host tissues where free arabinose is not available [31], *crp* and *fur* are no longer expressed and the cells become attenuated [28]. As expected for *Δ*P_crp527_::TT *araC* P_BAD_ *crp* mutants, all three RASTyV strains formed red colonies on MacConkey maltose agar in the presence of arabinose, and white colonies in the absence of arabinose [28]. The ΔP_fur81_::TT *araC* P_BAD_ *fur* phenotype was tested using CAS plates. In absence of arabinose, a yellow ring was observed around the colony, indicating siderophore production. In presence of arabinose, the indicative yellow ring was not observed as expected [28]. The OMP profile showed that the iron-uptake OMPs, repressed by Fur, were down regulated in presence of arabinose and up regulated in absence of arabinose (data not shown; see reference [29]). During strain construction we observed that a ΔP_fur33_::TT *araC* P_BAD_ *fur S*. Typhimurium mutant synthesized too much Fur to be optimally attenuated after growth in 0.2% arabinose [28]. We therefore replaced the ΔP_fur33_::TT *araC* P_BAD_ *fur* deletion/insertion with the ΔP_fur81_::TT *araC* P_BAD_ *fur* deletion/insertion, in which the ATG start codon of the *fur* gene was changed to GTG [28]. In *S*. Typhimurium ΔP_fur81_::TT *araC* P_BAD_ *fur* strains produce less Fur than ΔP_fur33_::TT *araC* P_BAD_ *fur* strains [28]. Arabinose-regulated synthesis of Crp and Fur in all three RASTyV was confirmed by western blot analysis (Fig. 2).

The phenotypes of ΔP_phoPQ107_::TT *araC* P_BAD_ *phoPQ* in the absence and presence of arabinose and PhoP^+^ strains were tested by 3XD plates supplemented with 50 µg/ml of 5-bromo-4-chloro-3-indolyl-phosphate to reveal acid phosphatase activity. Complementarily, the expression of PhoP was tested by western blot (data not shown). However, during the course of strain construction, we observed that a ΔP_phoPQ107_::TT *araC* P_BAD_ *phoPQ* mutant of *S*. Typhimurium is only moderately immunogenic, although highly attenuated [28]. We attempted several modifications of the construct to change the level of *phoP* expression, similar to the strategy we used to improve the *fur* construct, but none of the changes significantly improved the immunogenicity profile [28]. Therefore, we decided against using this mutation in our *S*. Typhi vaccine vectors. Rather than completely remake our three vector strains, we replaced the ΔP_phoPQ107_ mutation with the wild-type *phoP* promoter from *S*. Typhi ISP1820 in all strains (Table 1; Fig. 1).

The Δ*agfBAC811* mutation serves to reduce the bacterium's ability to form thin aggregative fimbriae [74] thus reducing its ability to survive and persist in the environment [75]. The Δ(*gmd-fcl*) deletion blocks the conversion of GDP-mannose to GDP-fucose, preventing colanic acid production [76], [77], which may contribute to biofilm formation *in vivo* [78]. The Δ*agfBAC811* and Δ(*gmd-fcl*) mutations were confirmed by PCR using the relevant primers shown in Table 3 (data not shown).

The *S*. Typhi Vi capsular polysaccharide is involved in immune evasion and has immunosuppressive effects on the host [79]. Also, the Vi antigen inhibits bacterial adhesion and invasion of intestinal epithelia [80]. TviA, a key regulator of Vi antigen synthesis, also represses expression of genes required for synthesis of flagella and the invasion-associated type 3 secretion system through repression of the flagellar regulators *flhDC* and *fliZ*, resulting in reduced invasion and reduced motility [81]. We deleted *tviABCDE*, genes required for the regulation and synthesis of the Vi capsule [82]. Wild-type strains with the Δ*tviABCDE10* deletion showed an increase in type 3 secretion when grown in LB broth (data not shown) and all three of the final vaccine strains were resistant to Vi-specific phage and did not agglutinate in presence of anti-Vi antiserum. Additional advantages for using a Vi^−^ strain in our constructs is that there will be no immune interference in individuals who have been vaccinated with Vi or Vi-conjugate vaccines and, along with the *pmi* deletion, serves to remove major surface antigens to decrease the host immune response against *Salmonella*.

The Δ*sopB1925* deletion was introduced because *sopB* mutations in *Salmonella* reduce fluid accumulation in ligated ileal loops [32] and enhance the host immune response against vectored antigens [35], [83]. The Δ*araBAD23* deletion was introduced into our *S*. Typhimurium vaccine strains to preclude acid formation due to arabinose fermentation during in vitro growth, as this leads to a cessation of growth [28]. Although *S*. Typhi does not utilize arabinose as carbon source, the *araBAD* sequences of *S*. Typhi Ty2 and *S*. Typhi CT18 are identical to the *S*. Typhimurium sequence. The *ΔaraBAD23* deletion was introduced into the ISP1820 derivative. No differences in arabinose utilization were detected on either MacConkey plates containing only arabinose or on minimal media containing arabinose as the sole carbon source, and therefore we did not introduce the *ΔaraBAD23* deletion into the *S*. Typhi Ty2 lineage.

Prior to the introduction of the *ΔasdA33* mutation, a functional *rpoS* gene from *S*. Typhi ISP1820 was introduced into the *S*. Typhi Ty2 derivative. The *ΔasdA33* mutation was then introduced into both the *S*. Typhi Ty2 and *S*. Typhi Ty2 RpoS^+^ derivatives, yielding three attenuated vaccine strains, two derived from Ty2, χ9639 and χ9640 (RpoS^+^), and one from ISP1820, χ9633 (Table 1; Fig. 1). The presence or absence of RpoS was confirmed by evaluating catalase activity and glycogen accumulation as described in the Materials and Methods. All strains had the expected phenotype: RpoS^+^ strains *S*. Typhi χ9640 and *S*. Typhi χ9633 were positive for catalase activity and glycogen accumulation and RpoS^−^ strains *S*. Typhi Ty2 χ9639 were negative for both phenotypes.

The API20E profiles of each of the RASTyV strains (containing pYA4088) were compared to the non-recombinant wild-type parents in the presence and absence of 0.2% arabinose. In the presence of arabinose, all three vaccine strains exhibited biochemical profiles consistent with Salmonella; API code 4104540 for χ9633(pYA4088) and 4004540 for χ9639(pYA4088) and χ9640(pYA4088). As expected, in the absence of arabinose, the profile for each strain was different, 4004000 for the Ty2 derivatives χ9639(pYA4088) and χ9640(pYA4088) and 4104000 for the ISP1820 derivative χ9639. The change in identification is the result of the lack of fermentation of all Crp-dependent sugars (*crp* is not expressed in the absence of arabinose). In the presence of arabinose, the API results for strain χ9633(pYA4088) deviated from the wild-type *S*. Typhi ISP1820 in respect to a positive reaction for ornithine decarboxylase (ODC). During the strain construction process, the positive ODC reaction was observed following the introduction of the ΔP_crp527_::TT *araC* P_BAD_ *crp* and ΔP_fur33_::TT *araC* P_BAD_ *fur* mutations. Neither deletion alone was sufficient to induce this phenotype: the phenotype was only observed when the mutations were combined and was observed in independently-derived strains, suggesting that this was not a spontaneous mutation elsewhere in the chromosome. However, it is unclear how the combination of ΔP_crp527_::TT *araC* P_BAD_ *crp* and ΔP_fur33_::TT *araC* P_BAD_ *fur* could result in synthesis of a functional ornithine decarboxylase (ODC) in *S*. Typhi ISP1820. While it is possible that a functional ornithine decarboxylase gene was introduced from S. Typhimurium through the transduction process used in strain construction, this is unlikely, as the ODC^+^ phenotype was only observed in ΔP_crp527_::TT *araC* P_BAD_ *crp* and ΔP_fur33_::TT *araC* P_BAD_ *fur* double mutants.

Type I fimbriae were synthesized in all three *S*. Typhi mutant strains when they were grown statically in LB broth containing 0.05% arabinose. Type I fimbrial synthesis was not assessed in the absence of arabinose, due to poor growth of the strains in arabinose-free media. LPS synthesis in all three vaccine strains was dependent of the presence of D-mannose in the media as expected due to the *Δpmi-2426* mutation (data not shown).

### Regulated protein synthesis in RASTyV strains

The Asd^+^ plasmid vector pYA4088 (Table 2), which complements the Δ*asdA33* deletion, carries DNA that codes for aa 3∼285 of the *S. pneumoniae* gene *pspA* [30], fused at the amino-terminal end to the β-lactamase type 2 secretion signal that directs PspA secretion out of the cytoplasm and into the periplasm and supernatant [6]. Plasmid pYA4088 and the parent plasmid, pYA3493 (Table 2) that does not carry *pspA* gene sequence [6], were introduced into the three *S*. Typhi vaccine strains. Plasmids pYA4088 and pYA3493 were maintained and stable in all strains for at least 60 generations. Each strain was grown in the presence of arabinose and then diluted into media without arabinose and serially passaged in arabinose-free media. Samples were taken after each passage and synthesis of proteins encoded by arabinose-regulated genes was assessed by western blots. We obtained similar results for all three strains. The results from strain *S*. Typhi ISP1820 χ9633(pYA4088) and χ9633(pYA3493) are shown in Figure 2A. As arabinose was diluted away, synthesis of Crp, Fur and LacI decreased, but PspA synthesis increased, as expected.

To verify PspA secretion, we fractionated cells from each strain and determined the level of PspA in each fraction. Our results indicated that greater than 50% of the PspA-Rx1 was found in the periplasm and supernatant for all three strains. Typical results, for *S*. Typhi ISP1820 χ9633(pYA4088) are shown in Figure 2B. These results are consistent with what has been reported for *S*. Typhimurium [6], [30].

### Sensitivity of RASTyV strains to transient deoxycholate exposure

**Figure 3:**
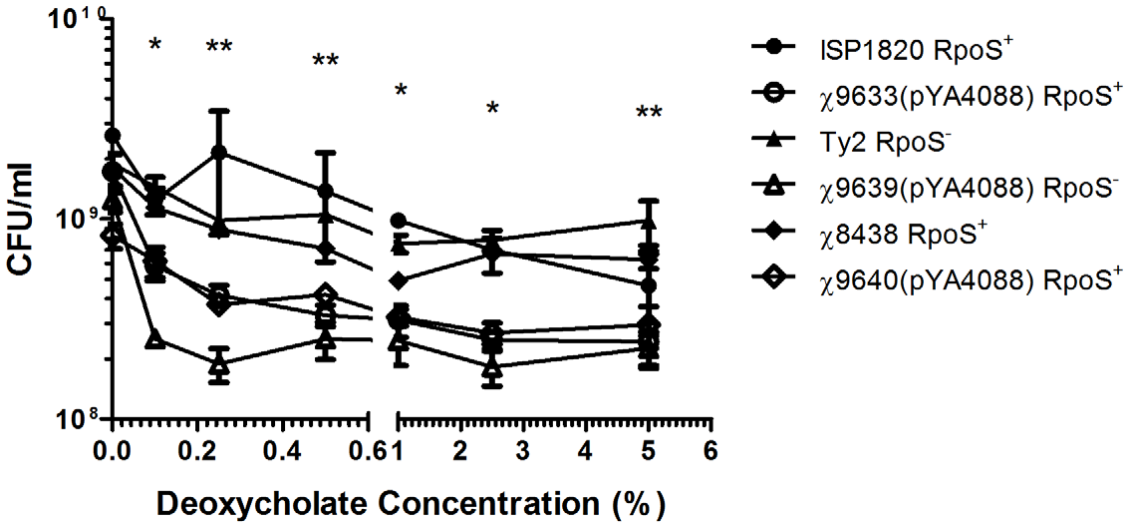
Sensitivity of RASTyV strains to transient deoxycholate exposure. *S*. Typhi strains (either RASTyV or wild-type) were diluted to a concentration of 1×10^9^ CFU/ml and transiently challenged with varying concentrations of sodium deoxycholate for 2 hours at 37°C. Survival of the *S*. Typhi strains following challenge was assessed by plating on LB agar + 0.2% arabinose. The Ty2-derived RASTyV strains were significantly more sensitive than wild-type to all concentrations of deoxycholate *, all parental strains different from their vaccine derivatives, *P*<0.05; ** 2 of the 3 parent strains different from their vaccine derivatives.

We noticed during the strain construction process that the RASTyV strains grew more slowly on bile-containing media such as MacConkey agar than the wild-type *S*. Typhi. This raised the possibility that one (or more) of the mutations introduced into the vaccine strains negatively impacted survival in the presence of bile, and thus the RASTyV strains might have difficulty surviving transit through the duodenum where bile from the gall bladder is introduced into the intestine. Therefore, we assessed survival of the RASTyV strains in the presence of deoxycholate compared to wild-type *S*. Typhi. Deoxycholate concentrations in the duodenum range from 0–1%, and may rise as high as 4% in the gall bladder [84]. The viability of all strains decreased as the amount of deoxycholate increased, but the behavior of the RASTyV strains differed depending on their parental background (Fig. 3). The *S*. Typhi Ty2-derived vaccine strain, χ9639(pYA4088) survived at a significantly lower rate than the wild-type *S*. Typhi at all concentrations of deoxycholate (*P*<0.05). The remaining RASTyV strains were significantly more sensitive to deoxycholate than the wild-type strains at most concentrations tested (*P*<0.05). However, for all vaccine strains, the decrease in resistance compared to wild-type resulted in a drop in cell numbers of less than 10-fold, suggesting that the vaccine strains should be able to transit the intestinal tract, but may not persist for long duration in the intestinal contents.

### RASTyV strain survival in chlorinated water, in sewage and untreated surface water

**Figure 4:**
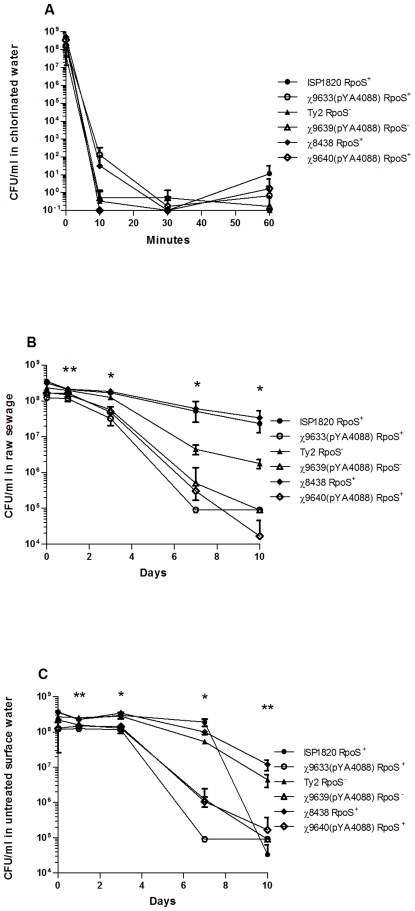
Survival of RASTyV strains in chlorinated tap water and raw sewage and untreated surface water. (A) Survival of RASTyV strains in chlorinated water. 1 ml of *S*. Typhi containing 1×10^9^ CFU was added to 19 ml of chlorinated water and viability was assessed by spread plating various dilutions at 0, 10, 30 and 60 min. (B) Survival of RASTyV strains in raw sewage. *S*. Typhi strains were inoculated into triplicate 20 ml sewage samples to a concentration of 1×10^8^ CFU/ml. Viability of the *Salmonella* strains was assessed on days 0, 1, 3, 7 and 10 after inoculation. (C) Survival of RASTyV strains in untreated surface water. *S*. Typhi strains were inoculated into triplicate 20 ml water samples to a concentration of 1×10^8^ CFU/ml. Viability of the *Salmonella* strains was assessed on days 0, 1, 3, 7 and 10 after inoculation. For B and C, *, all parental strains different from their vaccine derivatives, *P*<0.05; ** 2 of the 3 parent strains different from their vaccine derivatives.

In addition to being avirulent in the host, a live attenuated vaccine should possess some containment features to reduce its survival and persistence in nature to preclude vaccination of individuals who did not elect to be vaccinated [85]. We evaluated the survival of the RASTyV strains compared to their wild-type *S*. Typhi counterparts in chlorinated water, sewage and surface water, conditions designed to mimic the environment and to address concerns regarding the impact of releasing live attenuated, genetically-modified organisms into the environment. Since the chlorine concentration in our local tap water was found to vary significantly from day to day, we elected to mimic the chlorination process that occurs during municipal water treatment. All *S*. Typhi strains were extremely sensitive to chlorinated water experiencing several logs of killing after a 30-minute exposure (Fig. 4A).

In sewage, the titers of the RASTyV strains dropped steadily after one day, decreasing more than 3 logs in titer over the 10-day period (Fig. 4B). The wild-type strains survived significantly better than the vaccine strains (*P*<0.01). A similar trend was observed in the untreated surface water. The number of viable RASTyV bacteria decreased steadily after inoculation at a more rapid rate than the wild-type strains (Fig. 4C). The wild-type strains survived significantly better than the RASTyV strains (*P*<0.01).

### Survival of RASTyV strains in human blood, peripheral human mononuclear cells, and in guinea pig complement

**Figure 5:**
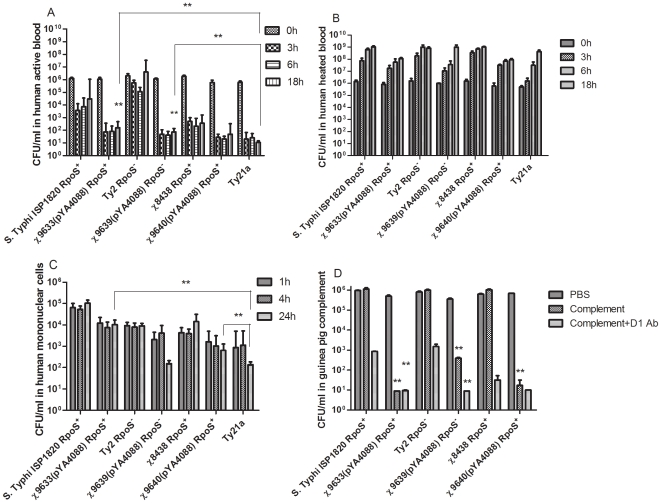
Survival of RASTyV strains in human blood, peripheral human mononuclear cells, and guinea pig complement. The bactericidal effects of (A) active whole blood and (B) heat-treated blood were compared by incubating 1×10^6^ CFU of each strain in 1.5 ml normal or heat-inactivated human whole blood. Bacterial survival was measured by spread plating at the indicated times after inoculation. **, *P*<0.01, for each RASTyV compared to its wild-type parent at 18 h. (C) *S*. Typhi survival in peripheral human mononuclear cells at 1 h, 4 h and 24 h after inoculation with 5×10^6^ CFU. **, *P*<0.01, for each RASTyV compared to its wild-type parent at 24 h, and significant differences between Ty21a and RASTyV are indicated. The assay was performed in duplicate and was repeated at least 3 times using blood from different individuals. The limit of detection was less than 10 CFU/ml. (D) *S*. Typhi survival in guinea pig complement three hours after inoculation with 1×10^6^ CFU. **, *P*<0.01, for the RASTyV strain compared to its wild-type parent at 3 h. Data shown are the arithmetic means of triplicate samples.

The development of rapid ex-vivo assays using human blood and freshly elutriated peripheral blood mononuclear cells (PBMCs) have been demonstrated as reliable tools for determining attenuation of *S*. Typhi for vaccine research and development [68], [86]. 1×10^6^ CFU of *S*. Typhi was added to duplicate 1.5 ml blood aliquots from volunteers. The numbers of all the RASTyV strains and Ty21a dropped below the detection threshold of 10 CFU/ml within 3 h and the numbers did not increase at later time points (Fig. 5A). In contrast, the wild-type *S*. Typhi ISP1820 and Ty2 parent strains survived significantly better than their vaccine derivatives χ9633(pYA4088) and χ9639(pYA4088), respectively (*P*<0.01). Surprisingly, the Ty2 RpoS^+^ strain χ8438 survived poorly in whole human blood. No killing was observed for any of the strains in heat-inactivated blood samples (Fig. 5B), indicating that the poor survival of the RASTyV strains is due to their inability to resist the microbicidal effects of active blood components.

An important part of *Salmonella* pathogenesis is the ability of the bacterium to survive and grow in macrophages [86], [87]. Therefore, we evaluated the ability of our strains to survive in human monocyte-derived macrophages (Fig. 5C). The three RASTyV strains and Ty21a were significantly reduced in their ability to persist and survive in PBMCs compared to their wild-type parents at 24 h after infection (*P*<0.01). The ISP1820 derivative, χ9633(pYA4088) survived better than any of the other vaccine strains, while the Ty2 derivatives, χ9639(pYA4088) and Ty21a were the most sensitive to killing.

Complement is an important blood component with bactericidal effects. We evaluated the relative sensitivities of the RASTyV strains and their wild-type counterparts to complement. Both wild-type and RASTyV strains were sensitive to antibody-dependent complement killing when incubated in the presence of anti-*Salmonella* O-antigen group D_1_ antibody (Fig. 5D). In all cases, the vaccine strains were more sensitive to complement than their wild-type counterparts (*P*<0.01). In the absence of *S*. Typhi-specific antibody, the wild-type strains were completely resistant to the effects of complement. The RASTyV strains remained sensitive to complement-mediated killing even in the absence of opsonizing antibody. This is consistent with the work done by R. Looney showing that the Vi capsule blocks C3 deposition on the surface of *S*. Typhi, and strains lacking Vi, such as the RASTyV strains used in this study, are susceptible to complement killing via the alternative (non-antibody) pathway [88].

### Distribution of *S*. Typhi strains in tissues of newborn mice

**Figure 6:**
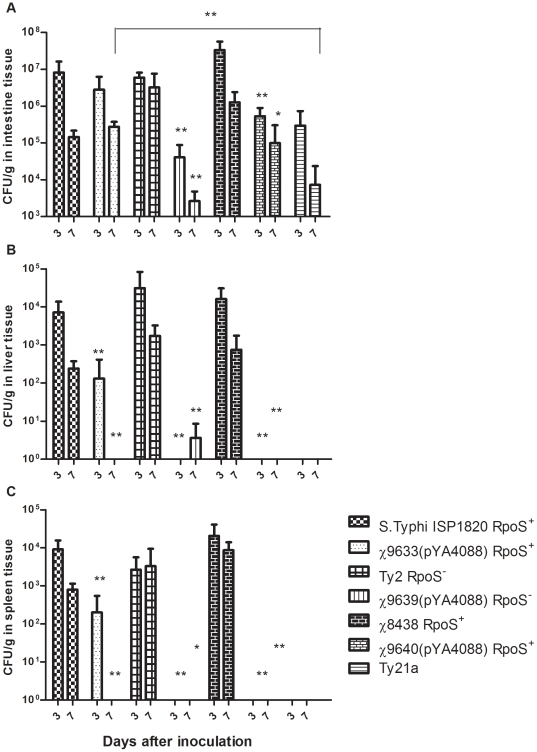
Distribution of RASTyV strains in tissues of newborn mice. The numbers of *Salmonella* bacteria in the intestines (A), liver (B) and spleen (C) at 3 and 7 days after oral inoculation of newborn mice with 1±0.2×10^9^ CFU of the indicated strains are plotted. Bars represent the arithmetic mean ± standard deviations from two separate experiments with 5 mice per group. *, *P*<0.05; **, *P*<0.01 for CFU counts in the indicated tissues for vaccine strains compared to their wild-type parent strains. The assays were performed twice.

Newborn mice (24 hours old) inoculated orally with 1±0.2×10^9^ CFU of RASTyV strains, wild-type parents or *S*. Typhi Ty21a survived without any symptoms of disease. We chose to use the oral route in these experiments to establish that our strains can overcome host defenses (e.g. low pH of stomach, presence of antibacterial substances in the gut) allowing it to invade the host mucosa and reach deeper tissues (e.g. spleen and liver). The bacterial burden in tissues of newborn mice were determined at 3 and 7 days post infection (Fig. 6). All strains were able to persist in the intestine for 7 days, although the Ty2 derivatives did so at significantly lower numbers than their wild-type parents (*P*<0.05) (Fig. 6A). All vaccine strains were less able to persist in the liver and spleen compared to the wild-type strains (Fig. 6B and Fig. 6C). The ISP1820 derivative χ9633(pYA4088) was present in both tissues on day 3, but was cleared by day 7.

### Antibody responses in mice after intranasal immunization with recombinant *Salmonella* vaccines

**Figure 7:**
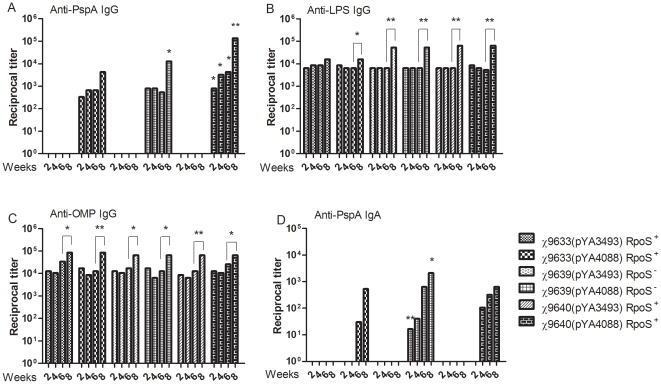
Immune responses against PspA, *S*. Typhi LPS and *S*. Typhi OMP in immunized mice. Serum IgG responses against rPspA (A), *S*. Typhi LPS (B), and *S*. Typhi OMP (C), and mucosal IgA responses to rPspA (D) were measured by ELISA using pooled sera and vaginal washes from BALB/c mice intranasally immunized with the indicated strains carrying either plasmid pYA3493 (control) or pYA4088 (PspA). Mice were boosted at week 6. ELISAs were performed twice with identical results. Significant differences were indicated *, *P*<0.05; **, *P*<0.01. No immune responses were detected to any antigen tested in mice immunized with only BSG or in preimmune sera from vaccinated mice (reciprocal titer <1∶50).

Groups of seven-week-old mice were intranasally immunized with 1±0.2×10^9^ CFU each test strain. All mice immunized with strains expressing *pspA* developed anti-PspA antibodies (Fig. 7A). Strain χ9640(pYA4088) (Ty2 RpoS^+^) induced significantly higher anti-PspA IgG titers than the ISP1820 derivative χ9633(pYA4088) at all time points (*P*<0.05 or *P*<0.01), and higher than the RpoS^−^ Ty2 derivative χ9639(pYA4088) at 4 weeks, 6 weeks and 8 weeks (*P*<0.05). Anti-PspA titers increased in all groups immunized with strains expressing *pspA* after the second immunization at 6 weeks. After this boost, anti-PspA IgG antibody titers in χ9639(pYA4088) immunized mice were significantly higher than in mice immunized with χ9633(pYA4088) (*P*<0.05). No anti-PspA IgG was detected in mice immunized with BSG (data not shown).

All attenuated *Salmonella* strains induced significant anti-LPS titers (Fig. 7B) as early as two weeks post inoculation. After the second immunization, significant boosting of serum antibody responses to LPS was observed (*P*<0.05 or *P*<0.01). Similar results were obtained for serum IgG responses against SOMPs (Fig. 7C).

Mucosal IgA anti-PspA responses were detected by week 2 in mice immunized with χ9639(pYA4088) (RpoS^−^), but not in mice immunized with the two RpoS^+^ strains (*P*<0.01) (Fig. 7D). By 4 weeks, anti-PspA IgA was detected in the χ9640(pYA4088) group. Statistical differences were seen between groups at each time point and titers reached a maximum by 8 weeks, after the boost that occurred at 6 weeks. IgA responses in mice immunized with χ9633(pYA4088) were the slowest to develop (Fig. 7D).

**Figure 8:**
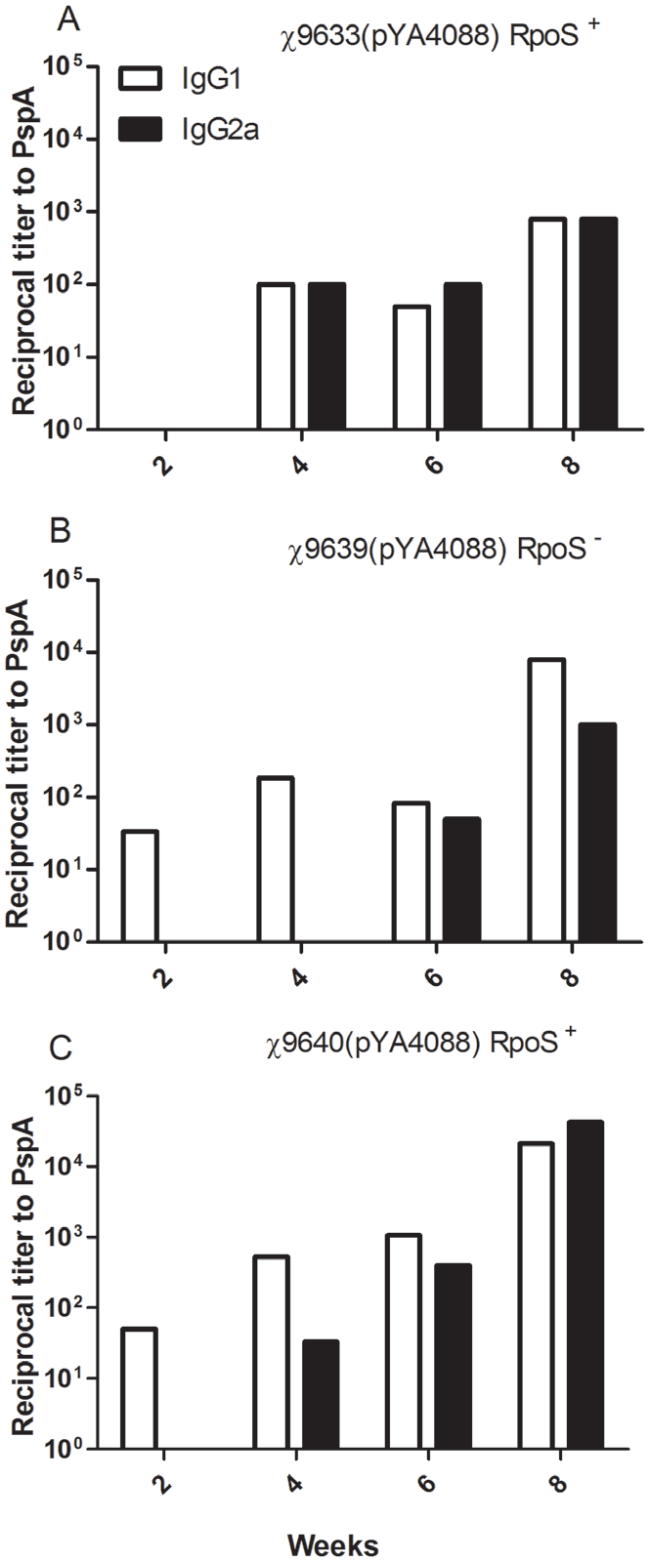
Serum IgG1 and IgG2a responses to rPspA measured by ELISA. Anti-rPspA IgG1 and IgG2a titers in pooled sera from BALB/c mice intranasally immunized with the indicated RASTyV strains at various times. Mice were boosted at week 6. The ratios of IgG1∶IgG2a at 8 weeks were 1∶1 for χ9633(pYA4088) (A) and χ9640(pYA4088) (C) immunized mice respectively; and 8∶1 for χ9639(pYA4088) (B) immunized mice. All ELISAs were performed twice with identical results.

The serum immune response to PspA was further examined by measuring the levels of IgG1 and IgG2a (Fig. 8). Mice immunized with ISP1820 derivative χ9633(pYA4088), developed a balanced Th1/Th2-type response against PspA (Fig. 8A), while mice immunized with the Ty2 derivatives initially developed a strong Th2-type response (Fig. 8B). Boosting with the RpoS^+^ strain χ9640(pYA4088) elicited a balanced Th1/Th2 response (Fig. 8C), while mice immunized with the RpoS^−^ strain χ9639(pYA4088) maintained a Th2-type response after boosting.

### Antigen-specific stimulation of IL-4 or IFN-γ production

**Figure 9:**
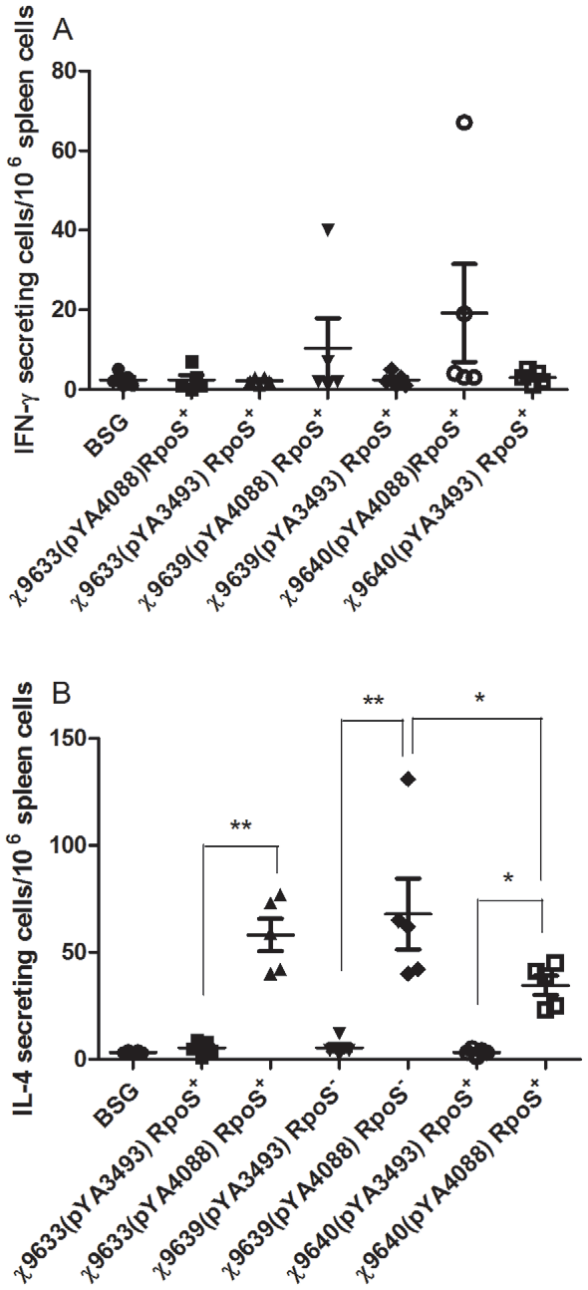
Antigen-specific stimulation of IFN-γ (A) or IL-4 (B). Splenectomies were performed on euthanized BALB/c mice 7 weeks after the primary immunization and one week after the boost. Buffer controls were also included. Splenocytes were harvested from 3 mice per group, and cells from each spleen were assayed in triplicate. Each symbol represents the results from a single well. The results from each well are presented as ELISPOTS per million splenocytes minus any background (≤4) ELISPOTS, from unpulsed mock controls. There were no significant differences between vaccine strains and the control group for secretion of IFN-γ. For secretion levels of IL-4, **, *P*<0.01 for χ9633(pYA4088) and χ9639(pYA4088) versus controls BSG group or empty vector group, *, *P*<0.05 for χ9640(pYA4088) versus BSG group or χ9640(pYA3493), and for χ9639(pYA4088) versus χ9640(pYA4088) as indicated.

To further evaluate the Th1/Th2 immune response, ELISPOT was used to determine numbers of IFN-**γ** (Th1-associated) and IL-4 (Th2-associated) secreting splenocytes from immunized and control mice in response to PspA (Fig. 9). Splenocytes from mice immunized with the RASTyV strains carrying pYA4088 produced very low levels of PspA-specific IFN-**γ** secreting cells (Fig. 9A). In contrast, splenocytes from the mice immunized with any of the RASTyV strains carrying pYA4088 produced significantly higher levels of PspA-specific IL-4 secreting cells than those of the empty vector controls (*P*<0.05 or *P*<0.01) (Fig. 9B). Of note, the results with strain χ9639(pYA4088), which induced a strong IL-4 response and an undetectable IFN-γ response, was consistent with the strong Th2 response indicated by the IgG1/IgG2a analysis (Fig. 9B).

### Evaluation of protective immunity

**Figure 10:**
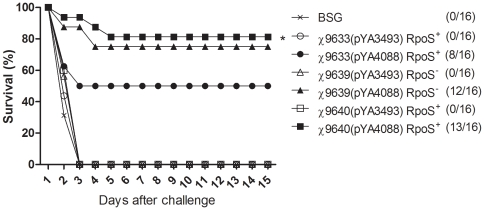
Evaluation of protective efficacy. Groups of eight 7-weekold mice were intranasally immunized twice at 6-week intervals with the indicated strains and challenged intraperitoneally with 1×10^4^ CFU of *S. pneumoniae* WU2 4 weeks later. The experiment was performed twice. Both experiments gave similar results, and the data have been pooled. The protection from each RASTyV strains was significantly different compared with control groups. *, *P*<0.05 for survival of mice immunized with χ9640(pYA4088) (Ty2 RpoS^+^) compared with survival of mice immunized with χ9633(pYA4088) (ISP1820).

To examine the ability of RASTyV strains expressing *pspA* to protect against pneumococcal infection, mice were challenged intraperitoneally with 1×10^4^ CFU of *S. pneumoniae* WU2 four weeks after the boost. *S. pneumoniae* WU2 produces PspA that is cross-reactive with PspA_Rx1_ encoded in pYA4088 [89]. All mice immunized with RASTyV expressing *pspA* were significantly protected compared with BSG and empty vector control groups (*P*<0.01) (Fig. 10). The protection afforded by the Ty2 derivatives, RpoS^+^χ9640(pYA4088) was significantly greater than that afforded by the ISP1820 derivative χ9633(pYA4088) (*P*<0.05).

## Discussion

Our ultimate goal is to design an RASTyV that synthesizes multiple pneumococcal antigens. One important often overlooked step in that process is to identify an optimal *Salmonella* vector strain. Therefore, we constructed, characterized and evaluated in mice three recombinant attenuated *S*. Typhi strains derived from ISP1820 and Ty2 (with and without a functional *rpoS* gene) synthesizing one antigen, the *S. pneumoniae* pneumococcal surface protein PspA, to evaluate which strain will make the best vector. One consideration in our strain design strategy was to test our hypothesis that immune responses induced to protective antigens will be superior in RpoS^+^ *S*. Typhi strains.

One of the most daunting problems encountered when evaluating an *S*. Typhi vaccine is making a pre-clinical determination of its safety and/or level of attenuation. Typically, an identical mutation is introduced into *S*. Typhimurium and the virulence of the strain is determined by oral administration to mice. While this has proven to be an accurate first approximation, it is clear that the interaction between *S*. Typhimurium and mice is not identical to the interaction between *S*. Typhi and humans. In some cases, attenuation of an *S*. Typhi mutant has been evaluated by injecting low doses of the vaccine strain intraperitoneally with hog gastric mucin into outbred mice [90]. This method has proven reliable for demonstrating the reduced virulence of a variety of *S*. Typhi strains that are attenuated by deletion or mutation of genes that result in a growth defect, such as *aro* mutants [91] and Ty21a [12]. However, the validity of this model is undermined by the observation that a *S*. Typhi *phoP* mutant, which has no growth defect, is just as virulent as wild-type strains [92] in this model despite the fact that both *S*. Typhi and *S*. Typhimurium Δ*phoPQ* mutants have been shown to be safe in Phase I clinical trials [18], [93]. In this study, we examined survival of the RASTyV strains in whole blood, in PMBC's and in the presence of complement as a surrogate for safety. Our strains were exquisitely sensitive to whole blood, with no detectable survivors after three hours (Fig. 5A). The vaccine strains were also more susceptible than their parent strains to killing by PBMCs (Fig. 5C), and by guinea pig complement (Fig. 5D). The ISP1820 derivative, χ9633(pYA4088), survived better in PBMCs than either of the Ty2 derivatives, which had similar survival curves. The survival profiles of our vaccine strains in whole blood and PBMCs was similar to the licensed typhoid vaccine strain Ty21a, indicating that their attenuation phenotypes resulted in a desirable safety profile.

Interestingly, strain χ8438, the wild-type Ty2 RpoS^+^ strain, had a survival profile in whole blood that was more like the RASTyV than wild-type Ty2. The reason for this is unclear, but it may be related to the fact that Ty2, isolated in 1918, has, presumably, been an *rpoS* mutant for many years. During that time, it is possible that it has acquired a number of suppressor mutations that enhanced its survival. Restoration of the mutant *rpoS* allele may have led to a phenotypically less fit strain, at least with respect to survival in blood.

This result does not appear to be related to Vi antigen production, since both *S*. Typhi χ8438 (Ty2 RpoS^+^) and *S*. Typhi ISP1820 make comparable amounts of Vi, although both strains make less Vi than Ty2 [48]. One difference between these strains that may account for these observations is that *S*. Typhi ISP1820 produces more LPS O-antigen than χ8438 [48], [82] and the production of long-chain O-antigen has been associated with complement resistance [94]. With regard to environmental safety, all strains were sensitive to chlorinated water and the RASTyV strains were less able to survive in raw sewage compared to their wild-type parents (Fig. 4). These results indicate that the RASTyV strains have a diminished capacity to persist in the environment.

In day-old mice, the three RASTyV strains exhibited different capacities for persistence of the intestinal tract. Higher numbers of RpoS^+^ strains *S*. Typhi χ9633(pYA4088) and *S*. Typhi χ9640(pYA4088) were isolated than of RpoS^−^ strain *S*. Typhi χ9639(pYA4088) (Fig. 6). Although our data are limited by the fact that we harvested the entire intestines, including contents, these results are consistent with the observation that RpoS regulated genes are necessary for invasion and colonization of M cells overlying the GALT [24], [26]. In spite of this shortcoming, the data indicate that an RpoS^+^ phenotype confers some advantage in the intestinal environment. All three of the wild-type strains were able to persist in the liver and spleen, while their attenuated counterparts were unable to maintain detectable numbers in these tissues. The inability of attenuated *S*. Typhi strains to persist in neonatal spleen and liver is consistent with a previous report [95] although in that study 7-day-old mice were inoculated intranasally. To our knowledge, this is the first report showing persistence in day-old mice with orally administered wild-type *S*. Typhi. We note that despite the ability of wild-type *S*. Typhi ISP1820, Ty2, and Ty2 RpoS^+^ to persist in neonatal mouse tissues, this does not lead to a lethal infection. (Santander, unpublished observation).

The three RASTyV strains stimulated different immune responses to PspA. The RpoS^+^ vaccine strains *S*. Typhi χ9633(pYA4088) and *S*. Typhi χ9640(pYA4088) induced mixed Th1- and Th2-type serum immune responses against rPspA and induced lower titers of IgA mucosal secretions than the RpoS^−^ vaccine strain *S*. Typhi χ9639(pYA4088). Despite the poor induction of IFN-γ and the comparatively strong induction of IL-4, all three strains induced high levels of PspA-specific serum antibodies (Figs. 7 and 9). Mice immunized with *S*. Typhi χ9640(pYA4088) or *S*. Typhi χ9639(pYA4088) produced the highest titers of PspA-specific serum antibody and experienced the highest level of protection, significantly greater than the protection seen in mice immunized with the ISP1820 derivative *S*. Typhi χ9633(pYA4088) (Fig. 10). It is not clear what the basis of these differences is and it is difficult to make predictions concerning which of the three strains will be the most immunogenic in humans, but it is clear that all three RASTyV strains are immunogenic and protective in the intranasal mouse model.

We have designed and fully evaluated three new RASTyV strains that include new features including regulated delayed in vivo attenuation and regulated delayed in vivo synthesis of the pneumococcal antigen PspA. We have demonstrated that these strains are immunogenic in mice, are highly susceptible to killing in human blood, and are poorly adapted for survival in the environment. Thus, the three RASTyV strains demonstrate a balance between a desirable safety profile and immunogenicity and should be completely safe when used in a clinical setting. The results from these preclinical studies pave the way for a phase 1 clinical trial, currently underway, where we are evaluating all three strains for safety and immunogenicity to determine the best *S*. Typhi vector to enable construction of an anti-pneumococcal vaccine. The clinical trial will allow us to address the importance of *rpoS* in RASTyV immunogenicity. Based on the results of the current clinical trial and ongoing work in our laboratory, we plan to make adjustments to the best of these three strains to further enhance safety to allow its use in younger and/or immunocompromised or malnourished populations and to incorporate additional protective pneumococcal antigens, with the goal of inducing protective immunity against all pneumococcal serotypes.
